# Valuing disaster risk reduction neighborhood interventions in informal settlements of Latin American and the Caribbean

**DOI:** 10.1371/journal.pone.0242409

**Published:** 2020-11-30

**Authors:** Alejandro Arrieta, Juan Pablo Sarmiento, Meenakshi Chabba, Weiwei Chen

**Affiliations:** 1 Department of Health Policy and Management, Florida International University, Miami, Florida, United States of America; 2 Extreme Events Institute, Florida International University, Miami, Florida, United States of America; 3 Department of Economics, Finance and Quantitative Analysis, Kennesaw State University, Kennesaw, Georgia, United States of America; University of Seville, SPAIN

## Abstract

This study assesses the dollar benefit of a neighborhood approach intervention on disaster risk reduction in small-sized, densely populated, and hazard-prone informal settlements across Latin American and Caribbean countries. We use a life satisfaction approach that assigns a dollar value to gains in wellbeing associated with the neighborhood approach intervention. Our primary data was a survey to a sample of 349 beneficiaries from small towns in Haiti, Guatemala, and Jamaica, and in major cities’ surrounded areas of Peru, Colombia, and Honduras. Out of 14 interventions, we found that community empowerment, physical works in public spaces and urban gardens/food approaches produced a gain of USD1,038 to USD1,241 to individual beneficiaries. Our study suggests a large benefit associated with the neighborhood approach intervention. It also shows that the life satisfaction approach is a promising method for the valuation of non-market and public goods, especially for countries where data on hazards and risks is not available to help monetize risk reductions.

## Introduction

Governments and humanitarian agencies strategize disaster risk reduction (DRR) interventions at multiple levels when confronting the various risks and vulnerabilities prevalent in hazard prone urban informal settlements. The interventions are often interwoven with development projects to achieve short-term gains in quality of life and long-term improvements in safety for the vulnerable at-risk communities. While these comprehensive interventions have the potential to improve the welfare of the communities, the methodological approaches to value their benefits are challenging. The complexity of the evaluation impact arises from the number of project elements implemented, and their multidimensional impacts that span social, economic, and environmental benefits. Standard economic methods to value these interventions, such as stated and revealed preference methods, are expensive and have several problems that limit its use [1–3]. An alternative method with growing applications in health, environmental, hazard, and disaster research [3–12] is the Life Satisfaction Approach (LSA), which is based on Hicksian compensation to identify the variation of income that would be necessary to offset the utility change produced by an intervention [1].

In this study, we use the LSA to assess the dollar benefit of the Neighborhood Approach (NA) strategy, a comprehensive DRR intervention that addresses multiple risks and vulnerabilities in small-sized, densely populated, and hazard prone urban informal settlements across Latin American and Caribbean (LAC) countries. Conceived and implemented by the United States Agency for International Development’s Office of U.S. Foreign Disaster Assistance (USAID/OFDA), the NA takes a broad look at neighborhoods and the complex web of interactions within to understand risk in informal urban settlements [13]. The NA intervention was implemented in six different LAC countries in partnership with different local nongovernmental organizations (NGOs) from 2012–2016 [14]. The NA interventions differed in the number of project elements they included from a set of 14 elements covering social mobilization, institutional arrangements, physical works, environmental improvements and financial mechanisms. Together, the project elements served to decrease vulnerabilities, empower communities, and build capacity, with the overall goal of achieving long-term disaster risk reduction [14]. Because of the multiple and different project elements included in the NA interventions, the value includes a wide range of overlapping benefits difficult to isolate.

The goal of this study is to estimate the benefit of each project element included in the NA interventions from the perspective of individual beneficiaries. Our contribution is twofold. First, we expand the economic evaluation literature of DRR to the case of interventions with multiple project elements in urban informal settlements. We conducted surveys in eight neighborhoods from six LAC countries to perform an economic evaluation of the NA intervention. Second, we use a new methodology based on the LSA to assess the contribution of each project element included in the NA interventions and assess its dollar benefit.

## Economic valuation of disaster risk reduction and the life satisfaction approach

### Economic value and methods to value DRR interventions

Concerned primarily with human well-being as its goal, the economic theory of valuation of a good or service measures its contribution to human well-being (utility) in monetary terms [15]. The monetary value of goods or services that cannot be traded in markets, such as public goods, can be estimated as the amount of money that would make an equivalent contribution to the utility of an individual [8]. This applies to DRR interventions that have characteristics of public goods [3,16]. They are non-excludable—once provided to a community, no one can be excluded from enjoying the benefits; they are non-rival—consumption of benefits by some does not diminish their availability to others. Economist have made significant inroads in valuing public goods and services in monetary terms using revealed preference and stated preference methods [2,15,17]. Revealed preference methods use observations of people’s real world behavior and choices to infer the value of public goods from market transactions in private goods [2]. Examples include the direct market prices and indirect methods such as the hedonic, travel cost, averting behavior, production function, etc. The stated preference method, as the name suggests, directly ask individuals to value public goods using hypothetical questions rather than real world choices. Methods include contingent valuation, contingent behavior, and conjoint analysis [2,15]. Although both methods are extensively and successfully used to estimate economic value to non-market and public goods and services, they do have limitations. Revealed preference methods rely on observed behavior which has been shown to be an incomplete measure of individual utility [3]. Stringent assumptions and difficulty in measuring non-values also mar the capacity of revealed preference methods. Meanwhile the hypothetical nature of stated preference methods often leads to criticisms of unreliability [2]. Both methods are also limited in their ability to capture the multidimensional benefits (which are also public goods) of development and mitigation projects such as the USAID/OFDA’s NA.

### The life satisfaction approach and subjective well-being

Similar to the stated preference and the reveled preference methods, the LSA also seeks to assess the economic value of a public good by finding the individual’s willingness-to-pay for it. However, instead of asking beneficiaries to think hard and assign a value to the good or service in question (state preference method), or eliciting a value from actual behavior in markets (reveled preference method), the LSA assess the willingness-to-pay by relating utility to both income and the consumption of public goods [1]. The LSA is based on the Hicksian compensating variation, or the amount of income that would need to be reduced to shift utility to the levels before the public good provision [18]. The method is used to measure both the marginal utility of the public good use and income, and then arrives at a trade-off ratio between the two. Suppose a DRR intervention reduces the probability of disaster risk in a community by 20% and a beneficiary individual expressed this change as an improvement in their life satisfaction by 1 unit. Simultaneously if an increase in income of $10,000 was also expressed by the individual as a life satisfaction increase by 1 unit, the LSA would equate the value of a 20% decrease in disaster risk at $10,000 (example adapted from Fujiwara and Campbell [8]). A growing number of LSA studies have been applied to DRR, including valuations of droughts [19], flooding [3,8], wildfires [20], air pollution [11,12], crime and health [4], value of life [5], chronic diseases [7], and terrorism [2].

Compared to traditional economic valuation methods, the LSA is a less restricted approach that relies on surveys of individuals’ subjective well-being (SWB) as adequate empirical approximation for individual utility [2,18]. SWB, defined as people’s emotional and cognitive evaluations of their lives, includes broad terms like happiness and life satisfaction [21]. Unlike contingent valuation, the LSA does not ask respondents to think hard and assign value to the good or service in question, it simply assess SWB by asking people to evaluate their happiness or rate their satisfaction in life without any explicit criteria [2], usually using the question “*Overall*, *how satisfied are you with life as a whole these days*?*”*[22]. The judgement is the individual’s alone. It is assumed that there is more reliability in people’s responses to how happy or satisfied they are with their life without knowing the reason behind it [23]. People tend to be more honest about a personal and subjective question as one on their life satisfaction and are likely to answer with instinct that nullifies strategic behavior [2]. The method uses a wider set of underlying assumptions as it does not rely on observed behavior, and thus allows the valuation of non-use values.

The LSA rests on the assumption that individuals seek to get the highest SWB. The pursuit of happiness by people and societies dates back to times immemorial, finding expression in Aristotle’s *Nicomachean Ethics* as happiness being, in and of itself, the ultimate goal in human existence [6]. This natural, innate desire for personal happiness—central to the human value system—gradually transformed into a collective goal for societies. Its importance was reiterated in 18^th^ century philosophical enlightenment thinking and by the 19^th^ century utilitarian creed that accorded the responsibility of individuals’ happiness to society [23]. Happiness as a collective goal inspired the 20^th^ century ideals of social reform and the development of the welfare state in the western world [23]. The objectives of welfare states in the developed world thus focused on public health, literacy and education, and food security (ending hunger) to construct the ideal society. As welfare states strengthened, they expanded their objectives to providing a good standard of living to its citizens mainly defined by monetary gains and income security [23]. Economic prosperity, measured by GDP per capita, became the primary pursuit of countries in the world.

The 1960s and 70s saw the western world experience a post materialistic phase and a disenchantment with traditional economic indicators that guided economic policy. This spurred extensive social science research in academia and government led entities to define social indicators to guide policy for social welfare. Several public opinion surveys like the Eurobarometer, and peer-reviewed literature laid the foundation for information and knowledge in the social indicator research field. From then on, social science research has use SWB to estimate happiness and individual welfare SWB [6,17]. Surveys of SWB have been applied in multiple cultures and countries [22]. In Latin America, SWB is included in public opinion surveys like the Latinobarometro [24] and LAPOP [25]. While studies on SWB in Latin America are limited [26], a pattern that arises in most studies is that Latin Americans enjoy very high life satisfaction that do not correlate with traditional socioeconomic and development indicators [27]. Compared to other regions, Latin America achieves higher SWB with lower materialistic values, stressing the importance of relational values (family ties and social relationships) to Latin Americans [27,28].

## Methods

This study was reviewed by the Institutional Review Board of Florida International University and received approval number IRB-17-0384. We used the LSA to assess the value of the NA intervention based on its impact on the life of beneficiaries. A SWB assessment is combined with income information to convert the effect of the intervention into a monetary figure [8].

Our approach used field surveys that assessed the SWB of 349 individuals (*i*) with residence in eight urban settlements benefitted by USAID/OFDA NA projects implemented from 2012–2016. The neighborhoods were located in small towns in Haiti, Guatemala, and Jamaica, and in major cities’ surrounded areas of Peru, Colombia, and Honduras, with direct beneficiaries that ranged from 2,300 to 116,000 residents. Each NA project consisted of up to 14 project elements P^j^ (*j* = 1,…,14) that are listed in Table 1. P^j^ is a binary variable that equals 1 if the project element *j* was part of the NA project in the corresponding neighborhood and equals 0 otherwise. Because the field surveys were conducted in 2017–2018, after the NA project implementation, we asked residents in the neighborhood to assess their current SWB and to recall their SWB before the NA project. Post-SWB was based on the answer to the question commonly used in the SWB literature [22], “*generally speaking*, *would you say you are satisfied with your life*?”, while pre-SWB was based on the answer to: “*You said you are [answer to post-SWB question] with your life now*. *What was your life satisfaction level before the project/intervention*?” The answers ranged from 1 (not at all satisfied) to 4 (very satisfied). The surveys also collected information on income and socio-demographic factors to incorporate the extensive literature showing that individuals with higher income, higher education and married usually have higher SWB levels [21,29].

**Table 1:** Descriptive statistics–individuals and project elements of the neighborhood approach intervention by neighborhood.

	Neighborhoods Intervened								
Variables	Rimac	Inde-penden-cia	Cara-bayllo	Mede-llin	Mixco	Port-de-Paix	Port-more	Tegu-cigalpa	Total
Post-SWB	3.41	3.29	3.36	3.30	3.41	2.88	2.90	3.59	3.27
ΔSWB	1.02	0.85	0.90	1.32	0.80	0.05	0.48	1.25	0.84
Annual income (in USD)	1,469	1,657	1,558	1,234	1,442	381	981	1,138	1,233
Years of school	9.4	8.6	9.0	7.5	5.2	3.8	10.8	6.6	7.6
Female (%)	70.5%	65.9%	76.2%	77.3%	61.0%	57.1%	66.7%	79.5%	69.4%
Age	44.6	50.4	35.8	35.5	40.8	45.2	51.1	39.3	42.8
Race-white (%)	2.3%	0.0%	2.4%	13.6%	0.0%	31.0%	0.0%	13.6%	7.9%
Race-indigenous (%)	4.5%	7.3%	0.0%	2.3%	19.5%	61.9%	0.0%	4.5%	12.4%
Race-black (%)	2.3%	2.4%	4.8%	11.4%	0.0%	7.1%	85.7%	0.0%	14.1%
Race-mestizo (%)	86.4%	90.2%	92.9%	72.7%	78.0%	0.0%	0.0%	81.8%	62.9%
Race-other (%)	2.3%	0.0%	0.0%	0.0%	0.0%	0.0%	2.4%	0.0%	0.6%
Marital status-married (%)	65.9%	65.9%	71.4%	63.6%	82.9%	42.9%	45.2%	45.5%	60.3%
Marital status-separated (%)	9.1%	14.6%	14.3%	4.5%	2.4%	7.1%	4.8%	15.9%	9.1%
Marital status-single (%)	25.0%	19.5%	14.3%	31.8%	14.6%	50.0%	50.0%	38.6%	30.6%
Project elements (P^j^)									
*Physical works*									
P^1^-Engineering and physical interventions	1	0	1	1	1	1	1	1	87.9%
P^2^-Public space	1	1	1	1	0	0	0	1	63.2%
*Social mobilization gains*									
P^3^-Capacity building	1	1	0	1	1	1	0	1	75.3%
P^4^-Community empowerment	1	1	1	1	1	0	0	1	75.3%
*Environmental improvements*									
P^5^-Environmental resilience	1	1	1	1	1	1	1	1	100%
*Institutional arrangements*									
P^6^-Governance	1	1	0	0	0	0	0	0	25.0%
P^7^-Regulatory Framework	0	1	1	0	0	0	0	0	24.4%
P^8^-GIS, information, and communication technologies	1	0	0	0	0	0	1	1	38.2%
*Livelihoods and Financial Mechanisms*									
P^9^-Markets and financing	0	0	1	1	1	0	1	1	62.6%
P^10^-Urban gardens/food	0	0	0	1	0	0	0	0	12.9%
P^11^-Urban livelihoods	0	0	0	0	0	1	0	1	25.3%
*DRR Intervention*									
P^12^-Early warning systems	0	0	0	1	0	1	0	1	38.2%
P^13^-Emergency and disaster management	0	1	0	1	1	1	0	0	49.4%
P^14^-Disaster Risk Reduction	0	1	0	1	0	0	0	0	25.0%

## Impact of project elements on SWB

Our goal was to estimate the contribution of each of the 14 NA project elements (P^j^) on the gain in SWB associated to the overall NA intervention, and then assign a dollar value to each project element based on the LSA. We achieve our goal in two steps. We first estimate a linear regression model to capture the contribution of an individual project element P^j^ on the total gain in SWB (ΔSWB), defined as the difference of post-SBW and pre-SWB:

$${\Delta SWB}_{i}=\beta_{o}+\beta_{j}P_{i}^{j}+\varepsilon_{i}$$

The parameter *β_j_* measures the individual contribution of project element *j* on SWB gain. The constant term *β*_0_ captures the contribution of all other project elements implemented in the neighborhood where individual *i* lives. The term *ε* represents the estimation error. Because individuals residing in the same neighborhood could be related by unobserved factors, we clustered standard errors by neighborhood. Notice that to identify *β_j_*, the project element *j* has to be implemented in some but not all NA interventions. Consequently, we cannot assess the value of project elements that were implemented in all neighborhoods, beneficing all individuals. In this study, that was the case of environmental interventions (P^5^), as described in Table 1.

### The dollar value of project elements

In the second step, we use the LSA by estimating the following linear regression model as it is traditionally used in the literature [1,2,17]:

$${postSWB}_{i}=\gamma ln(Y_{i})+\alpha X_{i}+u_{i}$$

The model estimates the association between post-SWB and individual annual income in logarithms–ln(Y)–, controlling for a set of socio-demographic variables–X–, including education (years of schooling), gender (female compare to male), age, race (white, indigenous and black compared to mestizo), and marital status (married and separated/divorced/widowed compared to single). We clustered standard errors by neighborhood. Because the post-SWB already includes the influence of the NA intervention, we assess the dollar value of the project element *j* based on the Hicksian compensation. Thus, we calculate the decrease in income (δY) necessary to shift the current individual utility (post-SWB) to pre-intervention levels, i.e. to the post-SWB minus the gain in SWB attributed to the project element *j* (parameter *β_j_* from the first step estimation):

$${postSWB}_{i}-{\hat{\beta }}_{j}=\hat{\gamma }ln(Y_{i}-\delta Y_{i})+\hat{\alpha }X_{i}$$

The dollar value per-person of the project element *j*, after averaging over all individuals, is calculated as $\delta \bar{Y}$, where $\delta ={1-e}^{\frac{\bar{postSWB}-\hat{\alpha }\bar{X}-{\hat{\beta }}_{j}}{\hat{\gamma }}-\bar{ln(Y)}}$. Because δ depends on estimated parameters, we produced standard errors for δ and δY using bootstrap resampling with replacement clustered by neighborhood.

## Results

Table 1 reports the descriptive statistics and implemented project elements in each neighborhood. The average post-SWB across all neighborhoods was 3.27, with an average SWB gain of 0.84 attributed to the NA interventions. Individual annual income ranged from USD381 in Port-de-Paix (Haiti) to USD1,657 in Independencia (Peru).

**Fig 1:**
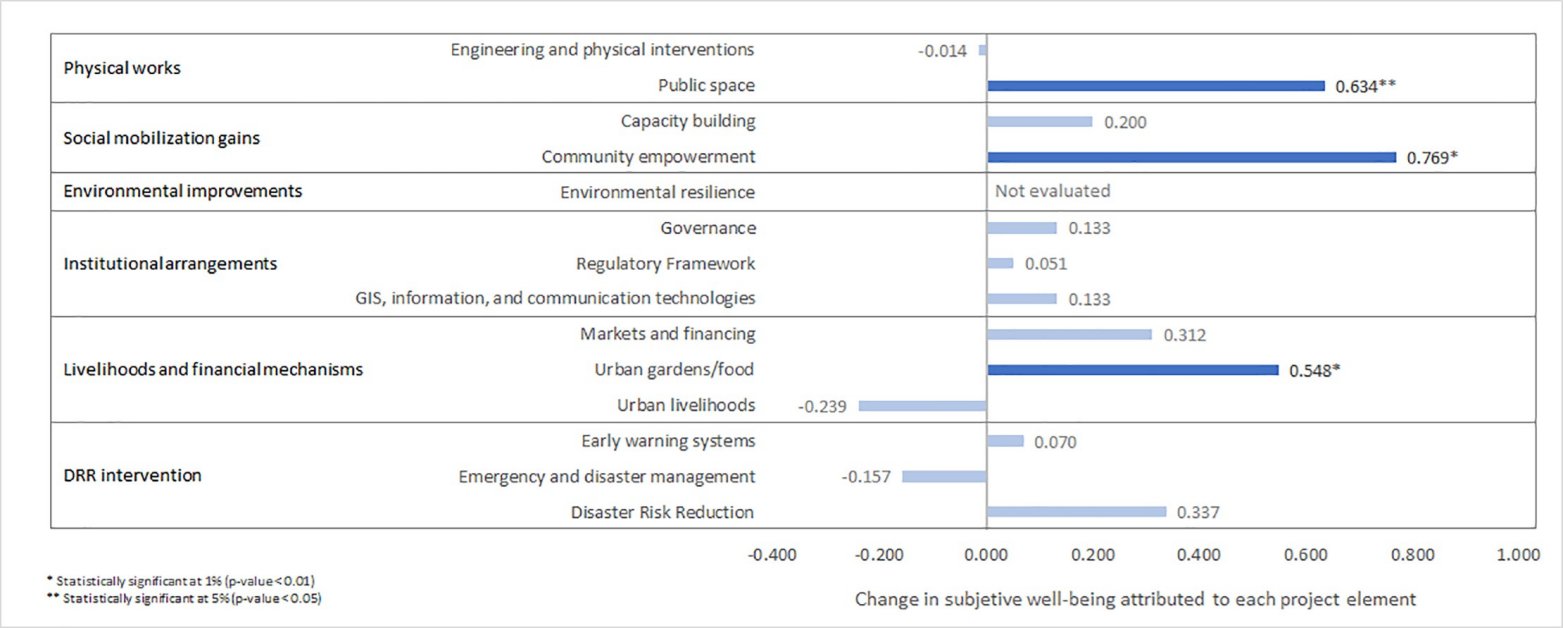
Impact of project elements on subjective well-being.

Fig 1 presents the results of the first-step estimation for all project elements grouped by categories. The categories with the highest impact on SWB gain are social mobilization gains and physical works. In particular, neighborhoods that received a community empowerment intervention (social mobilization category) increased their SWB by 0.769 points. Similarly, the physical works interventions on public spaces yielded a gain of 0.634 points in SWB for community beneficiaries.

Another project element that had a significant impact on SWB gain was the urban gardens/food approach (livelihoods and financial mechanisms). While in most cases the project elements were implemented in several neighborhoods (see Table 1), the urban gardens/food approach case with the positive impact corresponds only to the Medellin neighborhood so, a generalization of this case should be taken with caution.

Project elements such as GIS, information and communication technologies, capacity building, governance, regulatory framework, urban livelihoods, early warning systems, emergency/disaster management and disaster risk reduction were not statistically associated with changes in SWB.

The results of the second-step estimation are presented below. As expected, income has a statistically significant effect on SWB, as well as race and marital status. Higher income is associated with more SWB. Being married or separated has a positive contribution to SWB compared to single status. Being black is associated with lower SWB compared to other races.

$$\begin{array}{l} \mathrm{Post}‐SWB=0.120\;\ln(\mathrm{income})+0.002\;\mathrm{years}\;\mathrm{of}\;\mathrm{school}‐0.106\;\mathrm{female}‐0.002\;\mathrm{age}\\ \;(0.044)\;(0.013)\;(0.100)\;(0.002)\\ \;+0.011\;\mathrm{race}\;\mathrm{white}+0.231\;\mathrm{race}\;\mathrm{indigenous}‐0.499\;\mathrm{race}\;\mathrm{black}\\ \;(0.283)\;(0.155)\;(0.081)\\ \;+0.348\;\mathrm{married}+0.255\;\mathrm{separated}+2.393\\ \;(0.106)\;(0.136)\;(0.420)\\ R^{2}=0.121;\;\mathrm{MSE}=0.788 \end{array}$$

Based on the results of the two-step estimation, we obtain the dollar value of the project elements. Table 2 presents the results of those project elements that had a statistically significant impact on SWB. The valuation per direct beneficiary ranges from USD1,038 to USD1,241, equivalent to 82% to 98% of the average annual income of adults in the neighborhood. Using the estimated number of direct adult beneficiaries in all neighborhoods that received the corresponding intervention, the last column of Table 2 presents the dollar value of the total gain in SWB in the community. These values are only for adults (66% of the population in the neighborhoods), because SWB was not assessed for the young children and elderly population. Public space produced the largest gain in value, totaling more than USD 58.1 million. Community empowerment was the second most important project element, producing a value gain of USD53.2 million. The urban gardens/food approach produced a value gain of USD15 million.

**Table 2:** The dollar value of project elements.

Project element	Compensating income: δ% * (SE)	Valuation in 2017 USD		
		Per beneficiary ** (SE)	Direct adult beneficiaries	Total Value
Public space	97.9% (10.8%)	$1241.3 ($187)	46,790	$58,078,275
Community empowerment	88.7% (31.5%)	$1107.6 ($412.2)	48,085	$53,260,773
Urban gardens/food approaches	81.7% (37.8%)	$1038.4 ($496.9)	14,434	$14,988,569

* Corresponds to δ, the fraction of income that needs to be reduced to shift the current individual utility (post-SWB) to pre-intervention levels (see methods section).

** Corresponds to δY.

SE: Bootstrapped standard errors.

## Conclusions

We estimated the multidimensional public good benefits of the NA, a comprehensive strategy to reduce DRR in eight urban informal settlements across six LAC using people’s self-reported SWB. We used a novel methodological approach that identified the individual contribution of multiple elements of the NA intervention and assigned a dollar value to each element based on the LSA. Our results suggest that the NA intervention had a broad impact on the community well-being, with the largest improvements attributed to physical work on public spaces, social mobilization gains through community empowerment, and livelihood and financial mechanism in urban gardens/food approaches. The dollar value of gains in SWB attributed to those project elements ranged from USD1,038 to USD1,241 per direct beneficiary. While there are not similar projects to compare our results, studies on hazard preventions using the LSA valuation have found per-beneficiary willingness-to-pay ranging from $129 to avoid smoke-induced health effects of wildfire [20] to $6,505 to prevent a sure flood event [3] or $13,320 to avoid a drought [22].

There are important limitations to our study. First, our data was collected taking the family as the unit of analysis, but the SWB questionnaire was directed to an adult member of the family. Since the NA intervention may affect the SWB of each family member differently, our results could be biased. A more comprehensive approach should include SWB across different age groups or from a family perspective. Second, our survey was implemented post-intervention, and consequently the gains in SWB are estimated comparing neighborhoods with and without the intervention. A more robust approach should also compare neighborhoods before and after the intervention. Third, cost information of the NA project elements was largely missing, limiting our option for a cost-benefit analysis based on the LSA valuation. However, our results suggest that the USAID/OFDA NA projects could be cost-beneficial for the communities given the large benefits associated to improved life satisfaction.

A growing literature is using the LSA to assess the cost of natural hazards [3,8,20,22], but studies using the LSA to evaluate DRR projects are scarce. Our study shows a promising method that uses the LSA to value individual project elements of a comprehensive DRR intervention. From a practical perspective, our approach provides a low-cost alternative to traditional economic valuation methods. This is especially relevant for countries where data on hazards and risks is not available to help monetize risk reductions. Funders could easily incorporate our approach to evaluate the impact and cost-benefit of DRR interventions, and most importantly, to reallocate resources to the project elements that add more value to the overall DRR intervention.

## Supporting information

S1 File Minimal underlying dataset. (DOCX)
