# Supplementary material for: Valuing disaster risk reduction neighborhood interventions in informal settlements of Latin American and the Caribbean
Source: PLoS One. 2020 Nov 30;15(11):e0242409. doi: 10.1371/journal.pone.0242409 (PMC7703912; doi:10.1371/journal.pone.0242409)
Supplement: S1 File — (DOCX) [file pone.0242409.s001.docx]

**S1 File - Supporting Information**

**Minimal Underlying Dataset**

1. URL with de-identified dataset in Stata version 16.1:

[LSA_NAPublic.dta](https://fiudit-my.sharepoint.com/:u:/g/personal/alejarri_fiu_edu/ETEgvioNhE5NkzdFQUhF0KABS9AZYSILqKIlBd4BOvLJbA?e=QkYARB)

1. Variables in the dataset:

| **Variable label** | **Variable name** | **Value** | **Value label** |
| --- | --- | --- | --- |
| Neighborhood | ID_1 | 1 | Rimac |
|  |  | 2 | Independencia |
|  |  | 3 | Carabayllo |
|  |  | 4 | Medellin |
|  |  | 5 | Mixco |
|  |  | 6 | Port-de-Paix |
|  |  | 7 | Portmore |
|  |  | 8 | Tegucigalpa |
| Pre-SWB | ls0 | 1-4 |  |
| Post-SWB | ls | 1-4 |  |
| ΔSWB | ls_bef | -3,3 |  |
| Annual income (in USD) | yr_income |  |  |
| Years of school | yrs_school | 0-16 |  |
| Female | female | 1/0 | Female/Male |
| Age | age | 17-87 |  |
| Race | race_wht | 1/0 | White |
|  | race_ind | 1/0 | Indigenous |
|  | race_blk | 1/0 | Black |
|  | race_mez | 1/0 | Mestizo |
|  | race_ot | 1/0 | Other |
| Marital status | civ_married | 1/0 | Married |
|  | civ_separated | 1/0 | Separated |
|  | civ_single | 1/0 | Single |
| Project elements |  |  |  |
| Engineering and physical interventions | i1 | 1/0 | Yes/No |
| Public space | i2 | 1/0 | Yes/No |
| Capacity building | i3 | 1/0 | Yes/No |
| Community empowerment | i4 | 1/0 | Yes/No |
| Environmental resilience | i5 | 1/0 | Yes/No |
| Governance | i6 | 1/0 | Yes/No |
| Regulatory Framework | i7 | 1/0 | Yes/No |
| GIS, information, and communication technologies | i8 | 1/0 | Yes/No |
| Markets and financing | i9 | 1/0 | Yes/No |
| Urban gardens/food | i10 | 1/0 | Yes/No |
| Urban livelihoods | i11 | 1/0 | Yes/No |
| Early warning systems | i12 | 1/0 | Yes/No |
| Emergency and disaster management | i13 | 1/0 | Yes/No |
| Disaster Risk Reduction | i14 | 1/0 | Yes/No |
